# Comparison of coblation annuloplasty and radiofrequency thermocoagulation for treatment of lumbar discogenic pain

**DOI:** 10.1097/MD.0000000000008538

**Published:** 2017-11-27

**Authors:** Dongguang Sun, Quancheng Li, Yuanzhang Tang, Weiyi Gong, Liangliang He, Zhi Dou, Jiaxiang Ni

**Affiliations:** a Department of Pain Management, Xuanwu Hospital, Capital Medical University, Beijing, China; b Department of Pain Management, the First Affiliated Hospital of Harbin Medical University, Harbin, Heilongjiang Province, China.

**Keywords:** coblation annuloplasty, lumbar discogenic pain, radiofrequency thermocoagulation

## Abstract

This study aimed to compare the effectiveness and safety of coblation annuloplasty and radiofrequency thermocoagulation for lumbar discogenic pain.

Patients who suffered from lumbar discogenic pain and underwent coblation annuloplasty and radiofrequency thermocoagulation surgery were included. A questionnaire, including the visual analo scale (VAS), MacNab criteria, pain relief rate, and any complications due to surgery, was completed by the patients with the help of a trained volunteer who was blinded to the study. Data were collected at 1 week, and 1, 3, 6, and 12 months after surgery. Significant pain relief was defined as postoperative pain relief ≥50% compared with the preoperative state. Any complications during or after surgery were also recorded.

A total of 122 patients were included; 37 patients were lost in the follow-up and 85 were evaluated. Among these, 45 patients underwent coblation annuloplasty (CA group, n = 45) and 40 underwent radiofrequency thermocoagulation procedures (RF group, n = 40).

VAS pain scores were decreased at 1 week and 1, 3, 6, and 12 months postoperatively compared with preoperation in both groups (*P* < .05). The CA group had significantly lower VAS scores at 6 and 12 months of follow-up than did the RF group (*P* < .05). According to the modified MacNab criteria, the proportions of patients with excellent and/or good results at 3, 6, and 12 months of follow-up were significantly higher in the CA group compared with the RF group (*P* < .05).

Only 2 patients reported soreness at the needle insertion site in the CA group. However, 3 patients had soreness at the needle insertion site, 3 had increased intensity of low back pain, 1 had intracranial hypotension, and 2 had new numbness in the leg and foot in the RF group. At the 1-year follow-up, this numbness was present all of the time. No major complications occurred in the CA group.

Our study suggests that CA is a more effective and safe minimally invasive procedure than RF for treating lumbar discogenic pain.

## 1 Introduction

Lumbar discogenic pain accounts for more than 60% of patients who suffer from chronic low back pain and lower extremity pain.^[1]^ Pain lasting longer than 3 months is defined as chronic. If untreated, chronic pain can be debilitating, unrelenting, and severely affects quality of life. Socioeconomic consequences of low back pain are massive and a burden.^[2]^ Degenerative discs are responsible for lumbar discogenic pain,which is defined as a disorder of the nucleus pulposus, rupture of the annulus fibrosus, and injury of the cartilage endplate.^[3]^

There are some treatments for lumbar discogenic pain due to degenerative discs. Conservative noninvasive therapies include medication, physical therapy, never block, behavior management, and psychotherapy. However, recrudescence easily occurs and conservative treatment is sometimes ineffective in severe cases. Surgery is mainly used for severe cases, but complications, such as nerve root injury, epidural fibrosis, trauma to the cauda equina, vascular complications, and discitis, can occur.^[4]^ Over the past few years, a variety of minimally invasive percutaneous intradiscal techniques have been developed to minimize trauma, avoid injury of normal tissues, and improve recovery and clinical outcomes of patients.^[5]^

As a minimally invasive procedure, radiofrequency thermocoagulation technology is used to treat degenerative disc diseases by shrinkage of the prominent parts of the nucleus, denervation of the annulus, and thermocoagulation of inflammatory tissue.^[6,7]^ However, the surrounding tissue might be damaged by radiofrequency thermocoagulation at 70 to 90°C.^[8]^ Injury of the cartilage endplate by heat radiation and a change in spinal stability by contraction of the nucleus pulposus may accelerate degeneration of the intervertebral discs.

Since 1999, coblation nucleoplasty technology was approved for the spine by the US Food and Drug Administration. Coblation nucleoplasty not only has the above-mentioned 3 functions of radiofrequency thermocoagulation, but also has other characteristics, including ablation and a low temperature. The whole procedure works at relatively low temperatures (40–70°C), which decrease the risk of injury in the surrounding tissues.^[9]^ Many clinical studies have been performed to prove the efficacy of coblation nucleoplasty for low back pain with or without lower extremity pain secondary to contained lumbar disc herniation, and only a few complications have been reported.^[10–12]^ Coblation nucleoplasty technology overcomes the shortcomings of radiofrequency thermocoagulation technology.

However, the superiority of coblation annulopasty is not conclusive compared with radiofrequency thermocoagulation. Therefore, this study aimed to compare the effectiveness and safety of coblationan annulopasty and radiofrequency thermocoagulation in the treatment of lumbar discogenic low back pain and/or lower extremity pain.

## 2 Materials and methods

### 2.1 Patients

This retrospective study was approved by the Ethics Examining Committee of Human Research of Xuanwu Hospital, Capital Medical University, Beijing, China. A total of 122 patients with low back pain and/or lower extremity pain secondary to contained lumbar disc herniation underwent minimally invasive surgery during January 2013 to Octobor 2015 at Xuanwu Hospital.

The inclusion criteria were as follows: patients with low back pain and/or lower extremity pain for ≥3 months; age between18 and 70 years; Visual analog scale (VAS) score ≥4; failure of conservative therapy for longer than 6 weeks, including medication, physical therapy, and injection therapies (lumbar epidural injection and lumbarmedial branch block); contained disc herniation ≤6 mm and ≤1/3 of the sagittal diameter of the spinal canal and a disc height ≥50% compared with normal adjacent discs confirmed by lumbar magnetic resonance imaging; and positive provocation discography confirming concordant pain at the suspected disc.

The exclusion criteria were as follows: patients with major neurological deficits, such as sensory or motor deficits or asymmetrical reflexes; previous spinal surgery at the level to be treated; disc herniation with sequestration or disc space collapse; spinal fractures or tumors; spinal instability, spinal stenosis, or spinal deformity; infection; uncontrolled psychological disorders; and allergies to contrast media or drugs used in the procedure.

### 2.2 Procedure

The procedure was performed in an operating room with the patient in the prone position or in the lateral decubitus position. A 10-cm cushion was placed under the lumbar region. Cefazoline (1.5 g) was administered intravenously as a prophylactic antibiotic before the procedure. The patient was kept in the same position and received monitoring of vital signs. The puncture point was determined through the posterolateral approach toward the treat-disc level, which was approximately 8 to 10 cm lateral to the midline of the spine. After sterilization, the skin and soft tissues were infiltrated with local anesthetic. Under fluoroscopic guidance, a 17-guage cannula needle was slowly inserted into the outer annulus. The position of the cannula needle at the outer part of the annulus fibrosus was tested by loss of resistance with a glass syringe. Anteroposterior and lateral fluoroscopic views were also taken to confirm the position of the cannula needle at the outer part of the annulus fibrosus. Discography was performed with contrast material to evaluate annular integrity and concordant pain. The whole procedure was performed by a senior experienced pain physician.

### 2.3 Coblation annuloplasty

The coblation wand (UNITEC, China America United Technology [Beijing] Co. Ltd., China) was inserted into the cannula. The ablation function was approximately 5 mm beyond the tip of the cannula. This position was marked by a depth stop marker on the shaft of the wand. Movement or paresthesia in the patient's lower extremity was then tested with a coagulation radiofrequency controller set at 2 for half a second. Annuloplasty was achieved through 6 standard channels in the annulus. These channels were created by advancement of the wand in the ablation mode and retraction of the wand in the coagulation mode. After withdrawal of the wand, 2 mL of 0.5% lidocaine and 10 mg of triamcinolone acetonide were injected into the annuloplasty tract.

### 2.4 Radiofrequency thermocoagulation

An electrode was inserted into a 20-gauge 15-cm radiofrequency cannula (BMC, Montreal, QC, Canada) with a 10-mm active tip instead of a stylet. To avoid injury of nerves, electrical stimulation was carried out by motor stimulation at 2 Hz and sensory stimulation at 50 Hz. After 2 mL of 0.5% lidocaine was injected, radiofrequency thermocoagulation was performed at 70 to 90°C for 90 to 360 s. After withdrawal of the electrode, 2 mL of 0.5% lidocaine and 10 mg of triamcinolone acetonide were injected.

All of the patients were required to stay in bed for 48 hours following the procedure. After being discharged, patients were advised to perform limited walking, standing, and sitting. They were also advised to avoid lifting, bending, or stooping for 2 weeks. No strenuous activity was allowed for 3 months after the procedure.

### 2.5 Assessment of outcome

All of the patients were required to complete a questionnaire. Data were collected by a trained volunteer who was blinded to the study at 1 week and 1, 3, 6, and 12 months postoperatively.

Assessment of the VAS was performed by the patients themselves. A VAS score of 0 indicates no pain and a score of 10 indicates severe pain.

The modified MacNab criteria^[13]^ were used to assess the patient's functional status with outcomes of excellent, good, fair, and poor. Excellent results included disappearance of symptoms and complete recovery in working and life activities. Good results included occasional episodes of low back pain or leg pain, and no limitations in working and life activities. Fair results included symptom relief, but with limitations in certain working and life activities. Poor results included insufficient improvement of symptoms and requirement of periodic administration of drugs after surgical treatment. Excellent and/or good results were considered to be effective treatment, and fair and/or poor results were considered to be ineffective treatment.

Significant pain relief was defined as postoperative pain relief ≥50% compared with the preoperative state and was used to assess the patient's degree of pain relief. Complications, such as hemorrhages, discitis, worsening of pain, paresthesia, intracranial hypotension, numbness, and infection were recorded.

### 2.6 Statistical analysis

Data analysis was performed with SPSS 22.0 statistical package (SPSS Inc., IBM Corporation). For descriptive statistics, we used mean ± SD when the distribution of data was normal and used median (minimum − maximum) when the data were not normally distributed. Repeated measures analysis of variance (ANOVA) was used to compare the improvement in pain VAS scores between the preoperative and postoperative time points. Significant differences in changes in time between groups in terms of mean values were assessed with the independent *t*-test. The Wilcoxon signed-rank test was used to evaluate the functional status of patients after 12 months of follow-up. A value of *P* < .05 was considered statistically significant in all analyses.

## 3 Results

A total of 122 patients underwent radiofrequency thermocoagulation or coblation annulopasty surgery. After excluding patients who were lost in follow-up and those with incomplete data, a final total of 85 patients were evaluated. Coblation annuloplasty (CA group, n = 45) and radiofrequency thermocoagulation (RF group, n = 40) procedures were compared.

Demographic characteristics regarding sex distribution, age, duration of pain, preoperative VAS score, and the treated level are shown in Table 1. These characteristics were similar in both groups (*P* > .05).

**Table 1:**
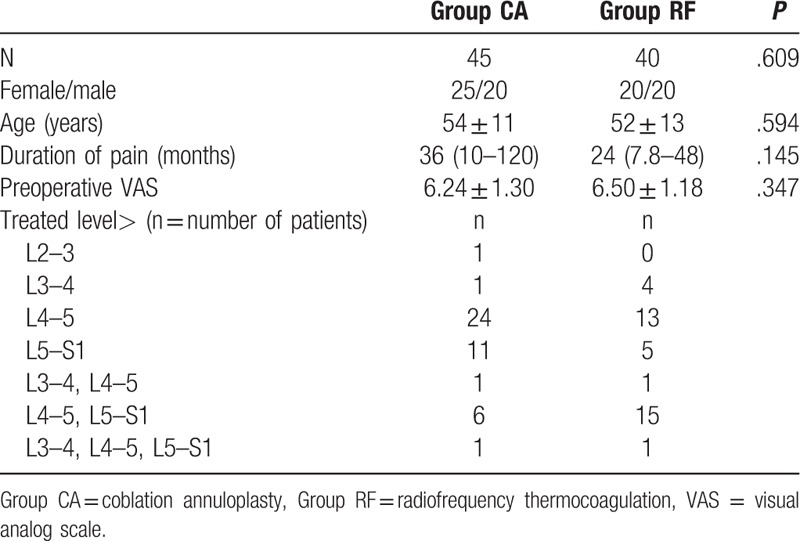
Demographic characteristic.

**Figure 1:**
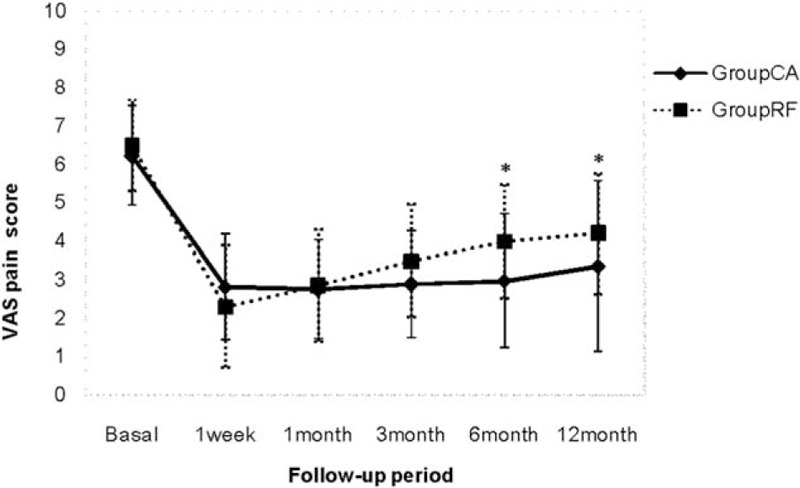
VAS pain scores were decreased at 1 week and 1, 3, 6, and 12 months postoperatively compared with preoperative in both groups. ^∗^ indicates significant difference in both groups. VAS = visual analog scale.

The VAS score was decreased at 1 week and 1, 3, 6, and 12 months postoperatively in both groups compared with preoperatively (Fig. 1). The VAS scores in the CA group at 6 and 12 months were significantly lower than those in the RF group (*P* < .05, Table 2).

**Table 2:**
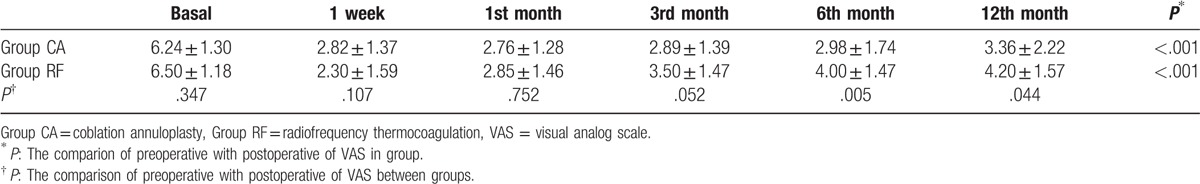
Assessment of VAS.

**Figure 2:**
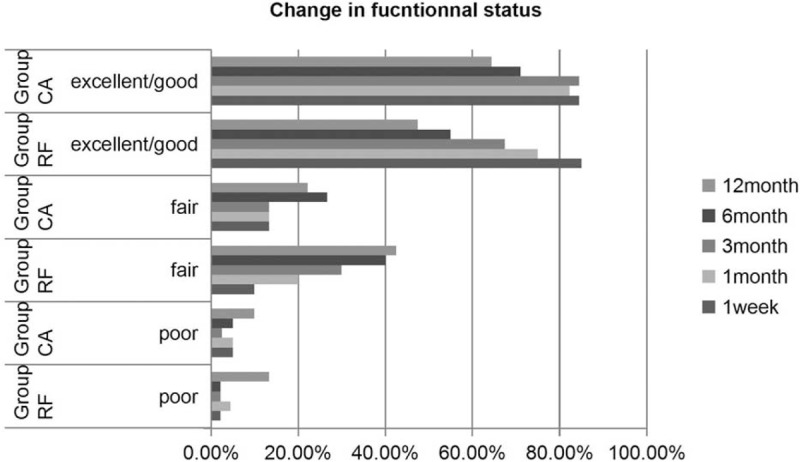
The proportions of patients with excellent and/or good outcomes after 3, 6, and 12 months were significantly higher in the CA group than in the RF group. CA = coblation annuloplasty, RF = radiofrequency thermocoagulation.

According to the modified MacNab criteria, the percentages of patients with excellent and/or good outcomes were 84.5%, 82.2%, 84.5%, 71.1%, and 64.4% at 1 week and 1, 3, 6, and 12 months in the CA group, and 85.0%, 75.0%, 67.5%, 55.0%, and 47.5% in the RF group, respectively (Table 3). The proportions of patients with excellent and/or good outcomes after 3, 6, and 12 months were significantly higher in the CA group than in the RF group (*P* < .05, Fig. 2).

**Table 3:**
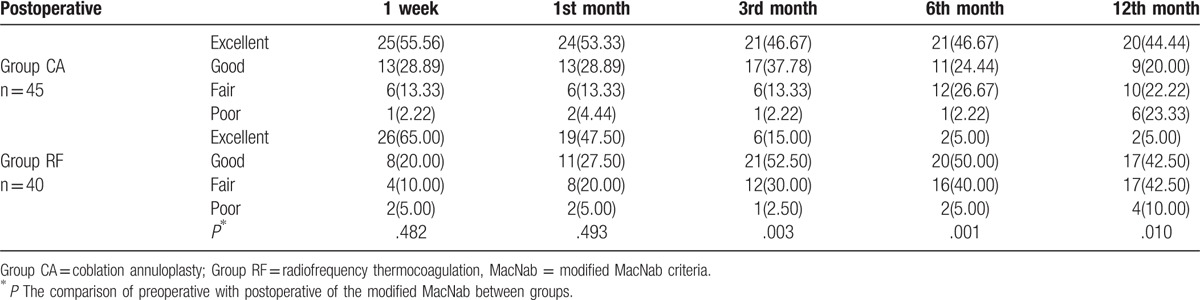
Assessment of modified MacNab.

**Figure 3:**
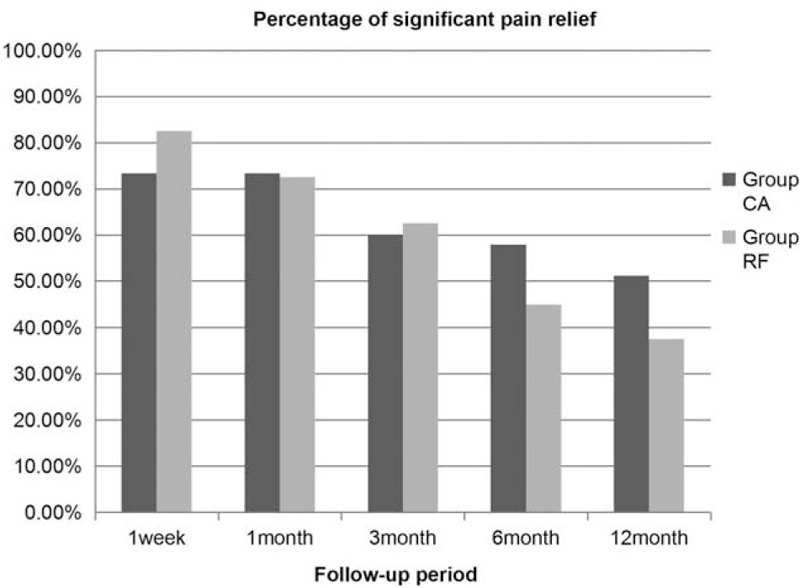
Percentages of patients who reported significant pain relief (postoperative pain relief ≥50% compared with the preoperative state) at 1 week and 1, 3, 6, and 12 months postoperatively in both groups. There were no significant differences in pain relief between the 2 groups.

A total of 33 (73.30%), 33 (73.30%), 27 (60%),26 (57.80%), and 23 (51.1%) patients reported significant pain relief at 1 week and 1, 3, 6, and 12 months postoperatively in the CA group, with 33 (82.5%), 29 (72.5%), 25 (62.5%), 18 (45%), and 15 (37.5%) patients in the RF group, respectively. There were no significant differences in pain relief between the 2 groups. (*P* > .05, Fig. 3).

In the CA group, 2 patients reported soreness at the needle insertion site, but the symptoms completely disappeared within 2 weeks after the operation. No hemorrhage, paresthesia, or infection was observed in the CA group. In the RF group, 3 patients reported soreness at the needle insertion site, 3 had increased intensity of low back pain, 1 had intracranial hypotension, and 2 reported new numbness in the leg and foot. At the 1-year follow-up, the numbness was present all of the time. However, there were no significant differences in complications between the 2 groups (*P* > .05, Table 4).

**Table 4:**
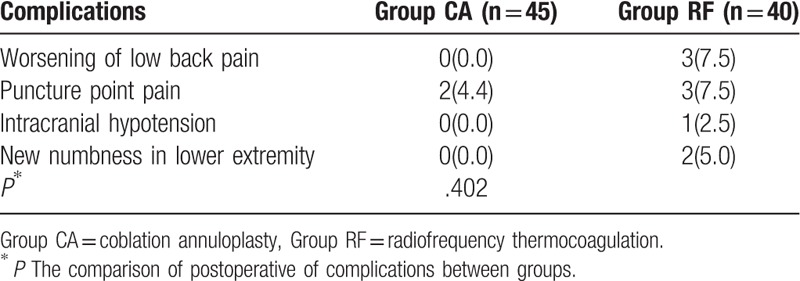
Complications related to the procedure.

## 4 Discussion

In recent years, radiofrequency thermocoagulation and coblation nucleoplasty have been widely used invasive techniques for discogenic pain due to degenerative discs. Few studies have compared the techniques of radiofrequency thermocoagulation and coblation nucleoplasty. In this study, the VAS pain scores were decreased at 1 week and 1, 3, 6, and 12 months postoperatively compared with preoperation in both groups. The CA group had significantly lower VAS scores at 6 and 12 months of follow-up than did the RF group. According to the modified MacNab criteria, the proportions of patients with excellent and/or good results at 3, 6, and 12 months of follow-up were significantly higher in the CA group compared with the RF group. However, there was no significant difference in pain relief between the 2 groups at 1 week and 1, 3, 6, and 12 months postoperatively. Less complications occurred in the CA group compared with the RF group, but this difference was not significant.

Few studies have focused on radiofrequency thermocoagulation, but its clinical application is wide. Radiofrequency thermocoagulation technology is used to treat degenerative disc diseases by shrinkage of the prominent parts of the nucleus, denervation of the annulus, and thermocoagulation of inflammatory tissue.^[6]^ Radiofrequency thermocoagulation is a minimally invasive percutaneous intradiscal technology, which is commonly used to treat low back pain and/or radicular pain. However, the clinical outcomes of this procedure are ambiguous. Manuel et al reported similar outcomes to those in our study in a study of radiofrequency for lumbar radicular pain in 25 patients. They found a decrease in the numeric rating scale score from 7.64 to 3.24 in 1 year and a decrease in the Oswestry Disability Index (ODI) score from 51.08% to 19.84% in 1 year. A total of 84% of patients who underwent radiofrequency achieved a significant level of satisfaction.^[7]^ However, a prospective, double-blind, randomized trial of 28 patients reported that percutaneous intradiscal radiofrequency thermocoagulation (90 s, 70°C) was not effective in reducing chronic discogenic low back pain.^[14]^ This study showed that the VAS score in the RF group decreased from 6.50 to 4.20, which is similar to Manuel et al's study. Therefore, RF is effective in the treatment of lumbar discogenic pain.

Clinical studies have shown the effectiveness of coblation nucleoplasty in treating low back pain with or without lower extremity pain secondary to contained lumbar disc herniation.^[10–12]^ In 2003, Singh et al^[15]^ reported that 75% of patients had a significant reduction in numeric pain scores at 12 months postoperatively compared with baseline, and 54% of patients showed pain relief of 50% or more at 12 months postoperatively. Additionally, significant improvement was reported in 54%, 44%, and 49% of patients in sitting, standing, and walking abilities at 12 months postoperatively, respectively. A retrospective study was conducted in 50 patients who underwent intradiscal coblation therapy for symptomatic contained lumbar degenerative disc disease. This retrospective study showed that analgesic consumption was reduced or stopped in 90% of patients after 1 year. Additionally, the VAS score was 4 points and the ODI was 7.2 compared with baseline at 24 months of follow-up.^[16]^

To date, only a few studies on annuloplasty for lumbar discogenic pain have been performed. In 2015, He et al^[17]^ reported the outcomes of coblation annuloplasty in 17 consecutive patients with discogenic low back pain. The preoperative pain VAS score was 6.5 ± 0.8. The pain VAS score decreased to 2.9 ± 1.6, 2.9 ± 1.7, 3.2 ± 1.6, and 3.2 ± 1.7 at 1 week and 1, 3, and 6 months postoperatively, respectively. According to the modified MacNab criteria, the proportions of patients with excellent and/or good ratings were 13 (76.5%), 11 (64.7%), and 10 (58.8%) at 1, 3, and 6 months of follow-up, respectively. No serious complications were observed. In this study, the preoperative and postoperative pain VAS scores, and the percentage of patients with excellent and/or good results were similar to those by He et al.

Nucleoplasty technology can be used for radicular pain.^[18,19]^ This procedure relieves pressure on the nerve roots by removing the nucleus pulposus, decreasing volume of the nucleus, and immediately reducing intradiscal pressure.^[20,21]^ Low back pain with or without lower extremity pain can be induced by pressure on nerve roots and ingrown nerve fibers in tears of the annulus fibrosus.^[17]^ Therefore, the effectiveness of coblation nucleoplasty in treating discogenic low back pain with or without lower extremity pain is uncertain. This is because the nerve fibers on the outer side of the fiber ring are not inactivated, and the fiber ring is considered as the major origin of discogenic pain.^[22]^ The target of our experimental treatment is more meaningful in the annulus fibrosus.

This study showed that CA had better results compared with RF. Improvement in the VAS score was greater in patients in the CA group than in the RF group. Additionally, the patient's functional status as determined by the modified MacNab criteria in the CA group was better than that in the RF group. The reason for this finding might be that RF action on the target, depending on the radiofrequency heat to contracture tissue, the electrode cannot be moved during the operation when the position of the cannula is confirmed. In CA, the wand can be advanced and retracted through 6 standard channels in the annulus, and the scope of work involved is greater than in RF. Other specific mechanisms need to be further studied.

In this study, 2 patients in the CA group reported soreness at the needle insertion site after the operation, but the symptoms completely disappeared within 2 weeks. No hemorrhage, paresthesia, or infection was observed. Three patients in the RF group reported soreness at the needle insertion site, 3 showed increased intensity of back pain, and 2 reported new numbness in the leg and foot. At the 1-year follow-up, this numbness was present all of the time. One patient suffered from intracranial hypotension. In 2011, Erdinc reported a burning-like sensation in the region on the lesion and an increase in severity of low back pain in 2 patients who were treated by RF. Radiofrequency lesioning of the medial branch interrupts the afferent and efferent neurons. This may be the reason and this may also be due to insufficient or partial denervation leading to neuropathic pain.^[23]^ The surrounding tissue might be damaged by heat radiation at 70–90°C.^[8]^ A temperature above the range of 42–50°C is cytotoxic to nerve fibers.^[24]^ Low intracranial pressure may also be due to dural injury caused by thermal radiation, resulting in cerebrospinal fluid extravasation.^[25]^ Nutrients in the intervertebral disc are transferred by the cartilage endplate.^[26]^ Injury of the cartilage endplate by heat radiation and a change in spinal stability by contraction of the nucleus pulposus may accelerate degeneration of intervertebral discs.

Some limitations of our study should be mentioned. Because of reasons involved in radiofrequency thermocoagulation technology, this technology has been eliminated in our department. Therefore, this study was not a prospective control study, which is a limitation. We found that bias was inevitable in the follow-up data. The study sample size was small. Further controlled studies with a larger sample size are necessary to better determine the efficacy of these treatments.

## 5 Conclusions

This study shows that coblation annuloplasty is a more effective and safe minimally invasive procedure than radiofrequency thermocoagulation. Coblation annuloplasty can be used as an alternative treatment for radiofrequency thermocoagulation for discogenic low back pain and/or lower extremity pain secondary to contained lumbar disc herniation.
